# Pyroptosis and Sarcopenia: Frontier Perspective of Disease Mechanism

**DOI:** 10.3390/cells11071078

**Published:** 2022-03-23

**Authors:** Hongfu Jin, Wenqing Xie, Miao He, Hengzhen Li, Wenfeng Xiao, Yusheng Li

**Affiliations:** 1 Department of Orthopedics, Xiangya Hospital, Central South University, Changsha 410008, China; 218112327@csu.edu.cn (H.J.); xiewenqing@csu.edu.cn (W.X.); hemiao@csu.edu.cn (M.H.); hengzhen@csu.edu.cn (H.L.); 2 National Clinical Research Center for Geriatric Disorders, Central South University, Xiangya Hospital, Changsha 410008, China

**Keywords:** sarcopenia, aging, NLRP, pyroptosis, gasdermin

## Abstract

With global ageing, sarcopenia, as an age-related disease, has brought a heavy burden to individuals and society. Increasing attention has been given to further exploring the morbidity mechanism and intervention measures for sarcopenia. Pyroptosis, also known as cellular inflammatory necrosis, is a kind of regulated cell death that plays a role in the ageing progress at the cellular level. It is closely related to age-related diseases such as cardiovascular diseases, Alzheimer’s disease, osteoarthritis, and sarcopenia. In the process of ageing, aggravated oxidative stress and poor skeletal muscle perfusion in ageing muscle tissues can activate the nod-like receptor (NLRP) family to trigger pyroptosis. Chronic inflammation is a representative characteristic of ageing. The levels of inflammatory factors such as TNF-α may activate the signaling pathways of pyroptosis by the NF-κB-GSDMD axis, which remains to be further studied. Autophagy is a protective mechanism in maintaining the integrity of intracellular organelles and the survival of cells in adverse conditions. The autophagy of skeletal muscle cells can inhibit the activation of the pyroptosis pathway to some extent. A profound understanding of the mechanism of pyroptosis in sarcopenia may help to identify new therapeutic targets in the future. This review article focuses on the role of pyroptosis in the development and progression of sarcopenia.

## 1. Introduction

With the global proportion of elders over the age of 65 increasing, it is estimated that this situation will worsen by 2050 with the proportion of elders exceeding 1.3 billion and reaching up to 38% of the population [1]. The ageing population poses a huge challenge to public health, placing a great burden on the country and society. Ageing is an important factor leading to increased susceptibility to disease and disability [2]. The occurrence of many diseases is related to ageing [3,4,5]. Studies have shown that 23% of the total burden of global diseases can be attributed to diseases from elders over the age of 60. The main factors of age-related disease burden are cardiovascular diseases (30.3%), malignant tumors (15.1%), chronic respiratory disease (9.5%), musculoskeletal disease (7.5%), and others (6.6%) [6]. Sarcopenia, an age-related disease, causes disability and declining quality of life in the elderly. Among the quality-of-life assessment tools, such as the EuroQol-5D instrument (EQ-5D) and Short-form General Health Survey (SF-36), the outcomes show that a higher proportion of quality-of-life problems are reported by patients with sarcopenia [7]. In addition to being affected by age, sarcopenia is also affected by genetic and lifestyle factors. Disease progression involves a decline in muscle mass and increased consequences, such as falling, disability, weakness, and increased mortality [8]. At present, an increasing number of researchers are focusing on the morbidity mechanism and intervention measures to prevent and treat sarcopenia.

In the process of aging, changes occur at the cellular level, such as autophagy, mitochondrial dysfunction, cell senescence, and DNA methylation [1,2,3]. All of these changes can occur in various cell types, causing damage to cell structure and function [4]. Pyroptosis, a new regulated cell death, was discovered recently, and is different from other cell death such as apoptosis, necrosis, and others in morphological characteristics, occurrence, and mechanism [5,6]. Pyroptosis mainly relies on inflammation to activate the inflammasome of the caspase family, leading to the activation of gasdermin proteins, then the activated gasdermin proteins transposition to the membrane, where the membrane is breached, causing cell swelling, cytoplasmic alterations, and ultimately leading to membrane rupture [7]. Studies have shown that pyroptosis is comprehensively involved in infectious diseases, atherosclerotic diseases, and age-related diseases [8,9,10]. Its role in sarcopenia is attracting more and more attention from researchers. Deeply understanding the role of pyroptosis in the development and progression of sarcopenia is helpful in providing new ideas for its clinical prevention and treatment. This review article focuses on the role of pyroptosis in the disease mechanism of sarcopenia.

## 2. Sarcopenia

With the ageing of the human body, there is inevitably a gradual decline in muscle mass, quality, and strength. Studies have shown that skeletal muscle mass and strength from the age of 40 decreases linearly, and the loss of skeletal muscle mass in individuals who are 80 years old can reach up to 50%, greatly impacting the elderly [11]. Since sarcopenia was first defined by Rosenberg, it has been used to describe the age-related loss of skeletal muscle mass and strength [12]. Research on sarcopenia continues to be a major focal point of research worldwide. However, there is a lack of a single diagnostic criterion for sarcopenia. The diagnosis of sarcopenia consists of the following three key characteristics: (a) muscle strength, (b) muscle quantity or mass, and (c) physical performance. Musculoskeletal degeneration not only impacts the daily mobility of patients but also leads to an increase in the incidence of complications, mortality, and morbidity in major surgical procedures [13].

The aetiology of sarcopenia is multifactorial, and both environmental factors and internal factors play a role in the onset of sarcopenia [14]. Decline in the activity of the elderly is one of the important reasons for sarcopenia. Studies have shown that exercise (especially resistance training) is the most promising method for increasing muscle mass and strength in elderly individuals [15]. Elderly individuals are often at risk of malnutrition, and protein-energy malnutrition, in particular, is often observed. Nutritional interventions may prevent and reverse the progression of sarcopenia [16]. The increased burden of chronic diseases in the elderly, such as chronic kidney disease and cirrhosis, leads to pain and disability, which causes sarcopenia known as secondary sarcopenia [16,17]. Abnormal muscle development and low birth weight are also implicated in the decline of muscle mass and strength in adulthood [18]. In vivo, inflammatory pathway activation, mitochondrial dysfunction, denervation, satellite cell reduction, and endocrine disorders are considered to be internal factors that cause sarcopenia [14]. Inflammatory cytokines have been shown to promote the loss of muscle mass by stimulating protein metabolism and inhibiting muscle anabolism. The results of a meta-analysis and systematic review showed that serum IL-6 and TNF-α levels in patients with sarcopenia were not significantly different from those in the control group, while the level of C-reactive protein (CRP) in serum increased [19]. Mitochondrial population changes and dysfunction are considered to be the key contributors to sarcopenia, leading to the production of reactive oxygen species (ROS) and promoting inflammation [20]. Age-related degeneration of neuromuscular junctions is associated with loss of skeletal muscle mass, which is a prominent aspect of sarcopenia. Endocrine disorders, such as low levels of 2,5-(OH) vitamin D and decreased levels of growth hormone (GH) and insulin-like growth factor-1 (IGF-1), have been observed in patients with sarcopenia. Studies have shown that testosterone can improve muscle mass, strength, and function, offering new ideas to develop possible interventions for sarcopenia. Satellite cells, also called muscle stem cells, are crucial for the muscle regeneration process. Satellite cells are reduced in the muscle tissues of patients with sarcopenia, suggesting that loss of satellite cells may be one of the pathogenic mechanisms of sarcopenia.

There is a link between apoptosis and sarcopenia. It is necessary to further clarify the apoptosis pathway related to sarcopenia. The increase in muscle protein decomposition is considered to be the result of the common effects of inflammation and sarcopenia [21]. A long-term low-grade inflammatory state is a significant feature of the ageing process, and involves concomitant chronic degenerative diseases, including sarcopenia. Several cytokines have been observed at high circulating levels, such as interleukin-6 (IL-6), C-reactive protein (CRP), and TNF-α [22]. TNF-α can induce apoptosis through a death receptor-mediated signaling pathway. Caspase-8 initiates caspase-mediated downstream cascade reactions, mediating crosstalk between the extrinsic and intrinsic apoptosis pathways [23]. The integrity of mitochondrial structure and function plays an important role in the clearance of mitochondrial oxidants, energy supply, protein repair, and degradation [23]. One possible consequence of mitochondrial dysfunction is the activation of apoptosis. Evidence has shown that mitochondrial-mediated apoptosis may be involved in the ageing process of skeletal muscle [24]. As a special form of apoptosis, pyroptosis occurs in aseptic inflammation, such as diabetes, atherosclerosis, acute liver injury, benign prostatic hyperplasia, Alzheimer’s disease and other diseases [25,26,27,28]. This review article focuses on the role of pyroptosis in sarcopenia.

## 3. Pyroptosis

### 3.1. Characteristics of Pyroptosis

Pyroptosis, a newly regulated form of cell death, was discovered and has been confirmed in recent years, and it is characterized by the release of a large amount of proinflammatory cytokines [29]. It is different from other kinds of cell death. Pyroptosis and necroptosis are both inflammatory death pathways that allow the release of immunogenic cellular contents, which may act as activators of pattern-recognition receptors (PRRs). However, they differ in morphological characteristics, occurrence, and regulatory mechanisms [30]. Necroptosis occurs when there are obstacles in normal apoptotic pathways. Pyroptosis always occurs after sensing potential destructive injury [7]. For example, the inflammasome involved in the pyroptosis pathway can be activated in injured tissue, metabolic macrophages, monocytes, and so on [31]. Pyroptosis can be induced by gasdermin, but necroptosis is induced by membrane-associated mixed lineage kinase domain-like (MLKL), resulting in distinct cell morphologies. Pyroptosis is also distinct from apoptosis, although they have similar characteristics in some ways. As an inflammatory mode of regulated cell death, pyroptosis has unique characteristics differing from apoptosis, such as intact nuclei, DNA laddering, pore formation, cell swelling, and osmotic lysis. There are also differences in the activation of the caspase enzyme; apoptosis mainly activates caspase-2, 6, 7, and 8, whereas pyroptosis mainly activates caspase-1, 4, 5, and 11. Poly (ADP-ribose) polymerase (PARP) cleavage and caspase-activated DNase inhibitor (ICAD) cleavage both exist in apoptotic cells; however, pyroptosis is unique [5].

### 3.2. The Canonical Inflammasome Pathway of Pyroptosis

Many extracellular stimuli (such as bacteria, viruses, toxins, among others) can induce pyroptosis [32,33]. In the canonical inflammasome pathway of pyroptosis, the inflammasome sensing protein nod-like receptor (NLRP), which has an N-terminal caspase recruitment domain (CARD)/pyrin domain (PYD), can be stimulated by foreign substances [34]. Members of the NLRP family consist of nod-like receptor 1 (NLRP1), nod-like receptor 2 (NLRP2), nod-like receptor 3 (NLRP3), and nod-like receptor C4 (NLRC4) [7]. NLRP3 can identify most extrinsic stimulations. When NLRP3 is stimulated, it is indirectly activated through K+ flow. Activated NLRP3 recruits an apoptosis-associated speck-like protein containing a caspase recruitment domain (ASC) adaptor, further forming an ASC focus by connecting CARD/PYD with ASC. Then, the ASC focus recruits pro-caspase-1, leading to the activation of caspase-1 [32]. Activated caspase-1 promotes the formation and activation of IL-18 and IL-1β [35]. Gasdermin D (GSDMD) plays an important role in the downstream signaling pathways in pyroptosis, and can form pores in the plasma membrane [36]. The destruction of the plasma membrane leads to the release of cytosolic proteins, causing cell swelling and cytolysis [37]. Caspase-1 can cleave GSDMD to release the membrane pore-forming GSDMD-N domain, promoting not only the release of proinflammatory cytokines, such as IL-18 and IL-1β, leading to inflammatory reactions, but also the process of pyroptosis [38]. NLRP1, unlike NLRP3, can only identify microbial muramyl dipeptide (MDP) [39,40]. Evidence has shown that NLRP2 is related to pro-caspase-1, which is involved in the production of IL-1β [41]. NLRC4 can mediate the activation of caspase-1 and pyroptosis events caused by Gram-negative bacteria, and its functions can be seen as a host defense strategy against pathogens. The activation of caspase-1 and pyroptosis events mediated by NLRC4 can promote the fusion of pathogen-containing phagosomes and lysosomes, leading to bacterial degradation [42]. Absent in melanoma (AIM)-like receptors can activate caspase-1 to induce pyroptosis in the same way as NLRP. For example, AIM2 can be activated, especially by double-stranded DNA (dsDNA), triggering pyroptosis signaling pathways [43].

### 3.3. Non-Canonical Inflammasome Pathway of Pyroptosis

The noncanonical inflammasome pathway is mediated by caspase-4/5/11. All of these proteins can cleave GSDMD, leading to the activation of pyroptosis signaling pathways [44]. First, caspase-4/5/11 can directly recognize cytosolic lipopolysaccharide (LPS) [45]. Then, the combination of caspase-4/5/11 and cytosolic LPS results in GSDMD cleavage, causing K+ efflux, which is sufficient to induce the formation of NLRP3 and activate the canonical pathway of pyroptosis [46]. Caspase-4/5/11-induced GSDMD pore formation can cause the release of proinflammatory cytokines and the occurrence of pyroptosis.

## 4. Pyroptosis and Sarcopenia

### 4.1. Activation of NLRP Family Triggers Pyroptosis

Local changes in muscle cell bioenergetics, mitochondrial metabolism, and oxidative damage play a role in the aggravation of sarcopenia [47]. Oxidative damage occurring in aged myofibers can trigger metabolic dysfunction, which may limit substrate availability for contractile performance [48,49]. The age-related loss of motor unit innervation can promote mitochondrial dysfunction, which is a vital cause of increased oxidative metabolism in muscle. Mitochondrial dysfunction is considered to be a major marker of the ageing process [50]. An efficient skeletal muscle energy supplement is derived from mitochondria. A large number of studies have shown that mitochondrial dysfunction and population changes play a role in the reduction of muscle mass [51]. Age-dependent decline in skeletal muscle mass is associated with muscle tissue metabolism [52]. Mitochondrial function is primarily involved in the production of ATP through oxidative phosphorylation (OXPHOS) but is also involved in apoptosis, calcium homeostasis, and the production of reactive oxygen species (ROS), reactive nitrogen species (RNS), and so on [51]. Moreover, oxygen radicals, such as ROS, can activate the NLRP family [53], which can propagate cellular metabolic dysfunction into immune responses. NLRP3-mediated processing of caspase-1 can cleave and activate IL-1β and IL-18, leading to activation of the canonical pathway of pyroptosis [36]. NLRP3-dependent caspase-1 activity increases with ageing, as has been found in mouse skeletal muscle. NLRP3 is also required for proteolysis and inactivation of reduced glyceraldehyde-phosphate dehydrogenase (GAPDH) and decreased glycolytic myofiber size during ageing in skeletal muscle. Animal studies showed that NLRP3 was a contributor to the age-related decline in myofiber size. However, there was no evidence that systemic inflammation was associated with the NLRP3-dependent decrease in glycolytic potential and myofiber size during ageing [54]. IL-1β is an important inflammatory factor that is positively correlated with age [55]. Studies have shown that age-related decline in muscle strength in the general population is closely related to the existence and duration of inflammatory conditions [56]. The ageing process of muscle is accompanied by chronic, low-grade inflammation, which has been found in skeletal muscle cells [57]. Released IL-1β has a strong proinflammatory effect, which further leads to the production of inflammatory molecules, such as IL-6, IL-8, and TNF-α [58]. These cytokines have been found at high levels in the blood serum of patients with sarcopenia, which may be related to the occurrence of sarcopenia [59]. TNF-α can promote protein degradation, reduce protein synthesis, and inhibit muscle regeneration by preventing proliferation and differentiation of satellite cells [60]. Studies on samples of skeletal muscle collected from cattle showed that the priming and activation of the NLRP3 inflammasome during ageing may trigger and sustain a proinflammatory environment leading to sarcopenia [61].

### 4.2. Lysosome-Induced Autophagy and Pyroptosis

Lysosomes, important organelles in skeletal muscle cells, are essential for cell homeostasis, and are the command and control center of cell signaling, metabolism, and other processes [62]. It is generally recognized that lysosomes play a key role in regulating cell death under both physiological and pathological conditions [63]. Mitochondria-derived products, such as ROS and NOS, can cause lipid peroxidation (LPO) in the lysosomal membrane, which can promote lysosomal membrane permeabilization (LMP) [64]. LMP is a key step in regulated cell death [65]. Lysosomes participate in regulated cell death in different ways, such as necroptosis, ferroptosis, and pyroptosis [66]. Under the triggering of various inducers, lysosomes become unstable and release their contents. Cathepsin B released by lysosomes is a central modulator of inflammasome-dependent pyroptosis [67]. Cathepsin B can not only bind to NLRP3 directly but also activate NLRP3 through mitogen-activated protein kinase (MAPK) and the transforming growth factor β-activated kinase 1 (TAK1)/c-Jun N-terminal kinase (JNK) pathway [68]. In addition to the release of the cathepsin family, lysosomal LMP results in ROS production and K^+^ efflux, leading to the activation of inflammasomes [69]. Therefore, rupture of the lysosomal membrane can be considered the upstream activator of NLRP, which can activate caspase-1 to trigger the canonical pathway of pyroptosis. LPS escapes the degradation of lysosomes, which allows LPS to activate the noncanonical pathway of pyroptosis.

**Figure 1:**
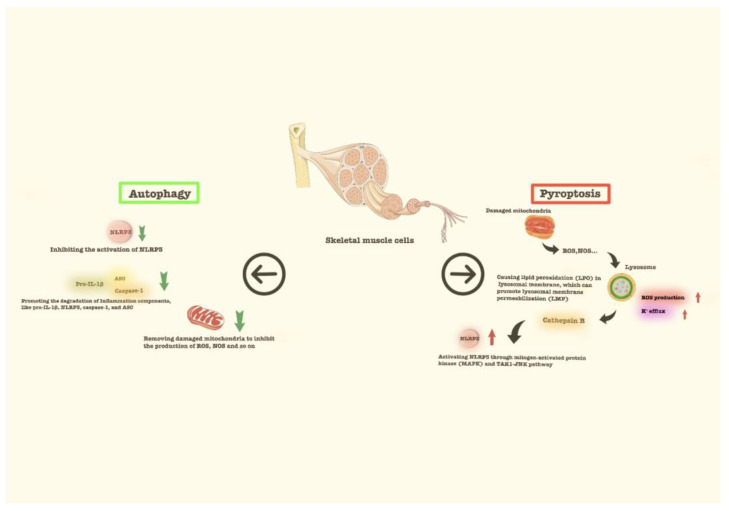
The autophagy of skeletal muscle cells in normal conditions can inhibit the activation of the pyroptosis to some extent. Autophagy can inhibit the activation of the nod-like receptor 3 (NLRP3) inflammasome by removing the damaged mitochondria, inhibiting the production of oxidizing agents, and promoting the degradation of inflammation components, such as pro-Interleukin-1β (pro-IL-1β), NLRP3, caspase-1, and apoptosis-associated speck-like protein containing a caspase recruitment domain (ASC).

As an important organelle, the lysosome also participates in autophagy, which is a protective mechanism in maintaining the integrity of intracellular organelles and the survival of cells in adverse conditions [70]. Studies have shown that autophagy can maintain muscle mass and enhance muscle strength to a certain extent [71]. Satellite cells, located between the basal lamina and the myofibrillar membrane, can regenerate and differentiate. Autophagy can activate satellite cells in response to muscle injury, leading to early compensatory regeneration of atrophic muscle [72]. When satellite cells are stimulated, they change from a resting state to an activated state with the ability to proliferate and differentiate [73]. With the ageing of skeletal muscles, stress from the imbalance of protein metabolism, increased oxidative stress and mitochondrial damage can lead to dysfunction of autophagy [74]. Autophagy can inhibit the activation of the NLRP3 inflammasome by removing damaged mitochondria to inhibit the production of oxidizing agents. Autophagy can also promote the degradation of inflammatory components, such as pro-IL-1β, NLRP3, caspase-1, and ASC [75] (Figure 1). All of these findings suggest that the autophagy of muscle cells can inhibit the activation of the pyrogenic pathway to some extent. Recently, studies have found that autophagy can also enhance the NLRP3 inflammasome, which in turn may cause pyroptosis [76]. Excessive activation of autophagy accelerates the decline in skeletal muscle mass [77].

### 4.3. Poor Skeletal Muscle Perfusion and Pyroptosis

Vascular dysfunction may become aggravated with increased age, and affects skeletal muscle perfusion, nutrient, and oxygen delivery to skeletal muscle, ultimately accelerating muscle mass loss and disability [78]. A positive correlation between the volume density of mitochondria and the number of capillaries supplying muscle fibers has been reported [79]. The number of capillaries supplying muscle fibers was also positively correlated with the size of muscle fibers [80]. The age-related decreases in fiber size are associated with capillary sparsity and oxygen-carrying capacity [81]. The heterogeneity of capillary spacing increases with age, and this seems to be related to the increased heterogeneity of muscle fiber size [82]. The distance between satellite cells and capillaries associated with type II fibers is longer in older people than in younger people, leading to the dysregulation of satellite cells and ultimately impairing muscle remodeling and regeneration [83]. Age-related biological processes such as oxidative stress, inflammation, and hormonal dysregulation can activate the NF-κB signaling pathway, increase P53/P21/P16 transcription, and inhibit autophagy. All of these factors may lead to dysfunction of vascular endothelial cells and vascular calcification. Vascular dysfunction may play a role in the development of sarcopenia [78]. ROS are mainly derived from endoplasmic reticulum (ER) stress, damaged mitochondria, and NADPH oxidase, and can trigger the activation of the NLRP3 inflammasome in endothelial cells, bridging the interaction between the NLRP3 inflammasome and endothelial dysfunction [84]. When the NLRP3 inflammasome is activated, it can further trigger endothelial cell pyroptosis, causing cell membrane rupture, cellular lysis, and the release of proinflammatory contents, including IL-1β, IL-18, and high-mobility group box 1 (HMGB1) [85]. IL-1β and IL-18, as the products of the NLRP3 inflammasome, can enhance the expression of inflammation-related genes of endothelial cells, such as adhesion molecules and chemokines, by activating the NF-κB pathway [86,87]. The inflammation-related pathological changes in the vascular endothelium may be involved in the early causative mechanisms in physical frailty and sarcopenia [88]. NLRP3 and ASC are phagocytosed by macrophages, leading to amplification of inflammation, suggesting that pyroptosis also plays a key role in transmitting inflammatory signals and amplifying the inflammatory reaction [89]. The loss of proper endothelial function may cause muscle tissue swelling, chronic inflammation, and the formation of thrombi [84]. Studies have shown that the dysfunction of vascular endothelial cells precedes a decline in muscle mass [90]. Vascular dysfunction can restrict blood flow and perfusion of skeletal muscle, blocking substrate transport to skeletal muscle and causing atrophy and dysfunction of skeletal muscle.

### 4.4. The NF-κB-GSDMD Axis Can Trigger Pyroptosis

**Figure 2:**
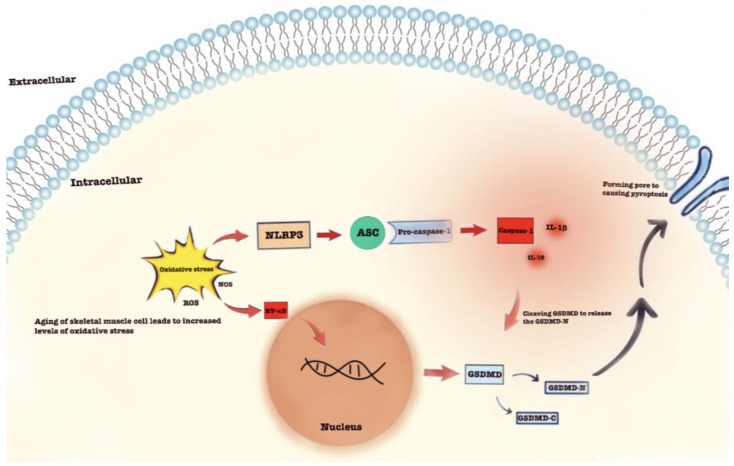
An increase in the level of oxidative stress caused by aging may trigger pyroptosis in skeletal muscle cells by the nuclear factor—kappaB (NF-κB)—gasdermin D (GSDMD) Axis. NF-κB is a transcription factor of GSDMD. When NLRP3 inflammasomes are activated by multiple activation mechanisms, the GSDMD will be subsequently cleaved, causing the release of the N-terminus of the GSDMD, which can form nanoscale pores in the cell membrane leading to the pyroptosis of skeletal muscle cells.

The development and maintenance of skeletal muscles require a variety of signaling pathways. In the relevant signaling pathways, nuclear factor kappa B (NF-κB) is a key factor in the maintenance of skeletal muscle homeostasis [91]. NF-κB exists in the form of a dimer and is involved in the occurrence and progression of various diseases related to inflammation, apoptosis, and proliferation [91]. In an unstimulated state, the nuclear localization sequence of NF-κB is bound to inhibitory IκB proteins. IκB kinase (IKK) can be stimulated by various stimuli, resulting in the phosphorylation of IκB and causing the degeneration of IκB. Then, the NF-κB complexes liberated from IκB can translocate into the nucleus to exert their actions [92]. Increased NF-κB signaling has been observed during ageing, and the concentration of NF-κB protein in the muscles of elderly people was found to be four times that of young people [93]. NF-κB is a key molecular switch in the cellular response to oxidative stress that can enhance the expression of the NLRP3 inflammasome and cytokines [94]. The levels of inflammatory factors in the blood circulation of elderly individuals are higher than those in young individuals [95]. As a key inflammatory cytokine produced by various cells, such as macrophages, lymphocytes, and fibroblasts, TNF-α acts as a central regulator to promote the expression of other inflammatory cytokines, which can significantly reduce the expression levels of myosin heavy chain (MHC) and the surface area of myotube cells and activate creatine kinase (CK), greatly impairing skeletal muscle function [96,97]. When TNF-α binds to TNF receptors R1/R2, NF-κB, signaling can be activated through the RIP1/TRAF2/IKK and TRAF2/NIK pathways [98,99]. Studies have shown that triptolide can prevent LPS-induced inflammation and skeletal muscle atrophy in mice by inhibiting NF-κB/TNF-α and regulating the protein synthesis/degradation pathway [100]. NF-κB is an important transcription factor of GSDMD. When extracellular pyroptosis-related signals activate NLRP3 inflammasomes, GSDMD is subsequently cleaved, causing the release of the N-terminus of GSDMD, which forms nanoscale pores in the cell membrane, leading to the release of proinflammatory substances and cell swelling [101]. Studies have shown that NLRP3 activation is associated with several age-related diseases [102,103]. Oxidative stress triggers the NF-κB-GSDMD signaling axis, which is the key pathway that regulates pyroptosis in inflammatory diseases and endothelial dysfunction [91]. The exact mechanism of the NF-κB-GSDMD axis in sarcopenia remains to be further studied (Figure 2).

## 5. Perspectives in Pyroptosis and Sarcopenia

Sarcopenia, a form of age-related disease, causes a gradual inability to maintain skeletal muscle function and quality [93]. With the gradual increase in the incidence of sarcopenia in the elderly population and health care expenditures, sarcopenia has now become the focus of research and public policy. Although the importance of sarcopenia is concerning, sarcopenia remains to be further understood. Pyroptosis, a special type of cell death, is mediated by activation of inflammasome sensors, including the NLR family, the DNA receptor absent in melanoma 2 (AIM2), and the pyrin receptor. Under normal conditions, inflammasome sensors detect a variety of pathogen-associated molecular patterns (PAMPs) and danger-associated molecular patterns (DAMPs) and respond to intracellular and extracellular danger signals. However, the unbalanced activation of this essential physiological sentinel function leads to aggravated pathological inflammation. The NLRP3 inflammasome is the inflammasome most closely related to uncontrolled inflammation [104]. The potential role of pyroptosis in skeletal muscle pathology has received special attention in recent years given that serious pathological changes in skeletal muscles can lead to weakness and disability. The NLRP family may be normally expressed in skeletal muscle, but its activation in the ageing process may help to enhance the adverse environment, changing muscle synthesis metabolism. Age-related increases in cytokines, such as IL-1β and IL-18, have shown activation of the NLRP family [61]. At present, the treatment of sarcopenia is mainly concentrated on physiotherapy, such as muscle strengthening and gait training and nutritional intervention [105]. No pharmacological intervention for sarcopenia currently exists [18]. The study of the pathogenesis mechanism of sarcopenia will help to identify new therapeutic targets in the future.

**Figure 3:**
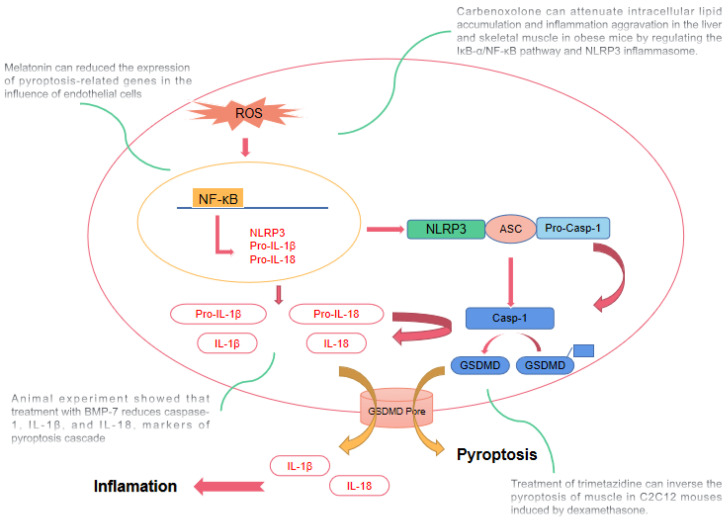
Possible interventions may focus on interfering with the signaling pathway of pyroptosis. a. Melatonin can reduce the expression of pyroptosis-related genes in the influence of endothelial cells. b. Treatment with bone morphogenetic protein 7 (BMP-7) reduces caspase-1, Interleukin-1β (IL-1β), and Interleukin-18 (IL-18), markers of pyroptosis cascade. c. Carbenoxolone can attenuate intracellular lipid accumulation and inflammation aggravation in the liver and skeletal muscle in obese mice induced by the high-fat diet by regulating the IkappaB-alpha (IκB-α)/ nuclear factor—kappaB (NF-κB) pathway and the nod-like receptor 3 (NLRP3) inflammasome. d. Treatment of trimetazidine can reverse the pyroptosis of muscle in C2C12 mouses induced by dexamethasone.

New interventions may focus on interfering with the signaling pathway of pyroptosis (Figure 3). Melatonin has significant anti-inflammatory characteristics, and an increasing number of people have begun to focus on the role of melatonin in sarcopenia [106]. Studies have found that melatonin also reduces the expression of pyroptosis-related genes, including NLRP3, ASC, lysed Caspase1, NF-κB/GSDMD, GSDMD N-terminal, IL-1β and IL-18, in endothelial cells. The potential therapeutic effects of melatonin need to be further verified [103]. Bone morphogenetic protein 7 (BMP-7), a drug commonly used to treat patients with osteoporosis, was recently found to alleviate diabetes-induced inflammation-mediated pyroptosis, sarcopenia, and adverse muscle remodeling. Treatment with BMP-7 reduces caspase-1, IL-1β, and IL-18, markers of the pyroptosis cascade [107]. Carbenoxolone (CBX) attenuates intracellular lipid accumulation and aggravation of inflammation in the liver and skeletal muscle in obese mice induced by a high-fat diet by regulating the IκB-α/NF-κB pathway and NLRP3 inflammasome, which can significantly reduce the expression of p-IκB-α, p-NF-κB, p-IRS-1, NLRP3, and inflammatory factors and increase the expression of p-PI3K and p-AKT [108]. Trimetazidine has been used as an anti-anginal agent for decades; however, recent evidence suggests that trimetazidine may also improve skeletal muscle performance in humans and mice [109,110]. Trimetazidine can protect skeletal muscle cells from starvation or inflammation-induced loss of mass by inhibiting protein degradation and inducing autophagy [111]. Studies have shown that treatment with trimetazidine can reverse the pyroptosis of muscle in C2C12 mice induced by dexamethasone, showing a protective role in skeletal muscle. Therefore, trimetazidine may be a potential therapeutic agent for the treatment of glucocorticoid-induced skeletal muscle disability. However, there is no evidence for its treatment of primary sarcopenia [112].

## 6. Conclusions

Sarcopenia, an age-related disease, can lead to a decline in skeletal muscle mass cause disability. With the gradual increase in the incidence of sarcopenia in the elderly population and health care expenditures, an increasing number of researchers are focusing on morbidity mechanisms and intervention measures to prevent and treat sarcopenia. Pyroptosis, also called inflammatory necrosis, may play a role in the mechanism of sarcopenia. In the process of ageing, aggravated oxidative stress and poor skeletal muscle perfusion in ageing muscle tissues can activate the NLRP family to trigger pyroptosis. Chronic, low-grade inflammation is a representative characteristic of the ageing process. The levels of inflammatory factors such as TNF-α may activate the signaling pathways of pyroptosis by the NF-κB-GSDMD axis, and this remains to be further studied. Autophagy is a protective mechanism in maintaining the integrity of intracellular organelles and the survival of cells in adverse conditions. The autophagy of skeletal muscle cells can inhibit the activation of the pyroptosis pathway to some extent. Study of the mechanism of pyroptosis in sarcopenia may be helpful for finding new therapeutic targets in the future.

## Data Availability

Not applicable.
